# Glucose-dependent phosphorylation signaling pathways and crosstalk to mitochondrial respiration in insulin secreting cells

**DOI:** 10.1186/s12964-019-0326-6

**Published:** 2019-02-20

**Authors:** Jaime Santo-Domingo, Antonio Núñez Galindo, Ornella Cominetti, Umberto De Marchi, Pedro Cutillas, Loïc Dayon, Andreas Wiederkehr

**Affiliations:** 1 Nestlé Institute of Health Sciences, Nestlé Research, EPFL Innovation Park Bâtiment G, 1015 Lausanne, Switzerland; 2 Analytical Signalling Group, Centre for Cell Signalling, Queen Mary University of London, London, UK

**Keywords:** Glucose, Mass spectrometry, Metabolism, Mitochondria, Signaling, Phospho-proteome, Kinase, Phosphatase, Beta-cell

## Abstract

**Background:**

Glucose is the main secretagogue of pancreatic beta-cells. Uptake and metabolism of the nutrient stimulates the beta-cell to release the blood glucose lowering hormone insulin. This metabolic activation is associated with a pronounced increase in mitochondrial respiration. Glucose stimulation also initiates a number of signal transduction pathways for the coordinated regulation of multiple biological processes required for insulin secretion.

**Methods:**

Shotgun proteomics including TiO_2_ enrichment of phosphorylated peptides followed by liquid chromatography tandem mass spectrometry on lysates from glucose-stimulated INS-1E cells was used to identify glucose regulated phosphorylated proteins and signal transduction pathways. Kinase substrate enrichment analysis (KSEA) was applied to identify key regulated kinases and phosphatases. Glucose-induced oxygen consumption was measured using a XF96 Seahorse instrument to reveal cross talk between glucose-regulated kinases and mitochondrial activation.

**Results:**

Our kinetic analysis of substrate phosphorylation reveal the molecular mechanism leading to rapid activation of insulin biogenesis, vesicle trafficking, insulin granule exocytosis and cytoskeleton remodeling. Kinase-substrate enrichment identified upstream kinases and phosphatases and time-dependent activity changes during glucose stimulation. Activity trajectories of well-known glucose-regulated kinases and phosphatases are described. In addition, we predict activity changes in a number of kinases including NUAK1, not or only poorly studied in the context of the pancreatic beta-cell. Furthermore, we pharmacologically tested whether signaling pathways predicted by kinase-substrate enrichment analysis affected glucose-dependent acceleration of mitochondrial respiration. We find that phosphoinositide 3-kinase, Ca2+/calmodulin dependent protein kinase and protein kinase C contribute to short-term regulation of energy metabolism.

**Conclusions:**

Our results provide a global view into the regulation of kinases and phosphatases in insulin secreting cells and suggest cross talk between glucose-induced signal transduction and mitochondrial activation.

**Electronic supplementary material:**

The online version of this article (10.1186/s12964-019-0326-6) contains supplementary material, which is available to authorized users.

## Background

Pancreatic beta-cells secrete the blood glucose lowering hormone insulin. Following a meal, glucose rises in the blood and acts as the primary secretagogue for insulin secretion [1]. Insulin secretion is biphasic. The first phase lasts several minutes and is mainly due to the exocytosis of a readily releasable pool of insulin granules [2]. During the second phase, insulin secretion increases gradually. This can take up to several hours when glucose remains elevated [3]. Therefore, during continued glucose stimulation beta-cells are able to adjust insulin biosynthesis and release to restore glucose homeostasis.

The beta-cell senses glucose through its uptake and metabolism rather than via a plasma membrane glucose receptor. Initial beta-cell activation is rapid as it takes less than 2 min for glucose to raise cytosolic calcium signals that trigger insulin granule exocytosis [4, 5]. The link between glucose metabolism and insulin secretion has been termed metabolism-secretion coupling. Mitochondria are essential for this process as they generate metabolic signals, including ATP, that control beta-cell electrical activity and associated calcium signals resulting in insulin exocytosis [6, 7]. Mitochondrial activation by glucose is a two-step process. First, glucose provides pyruvate, which rapidly accelerates mitochondrial oxidative metabolism and ATP synthesis [4, 8]. This early activation (< 2 min) initiates cytosolic and mitochondrial calcium signaling, which further accelerates respiration and mitochondrial ATP synthesis [9–11]. This slow calcium dependent rise in oxygen consumption lasts longer (> 30 min) and is required to assure sustained beta-cell activation [10, 12].

The transition from a resting to an activated beta-cell is not restricted to accelerated metabolism and insulin secretion. Several other biological processes undergo pronounced glucose-induced changes. For example, stimulatory glucose concentrations cause a general increase in protein biosynthesis through the activation of translation initiation [13, 14]. This is particularly true for preproinsulin where glucose stimulation stabilizes the mRNA and specifically activates translation of preproinsulin and other components of secretory granules (reviewed in [15]). Furthermore, glucose stimulates microtubule dynamics [16] and induces polymerization of the subplasmamembrane actin cytoskeleton [17]. By controlling cytoskeleton dynamics, glucose is able to promote the transfer of insulin granules from a reserve to a readily releasable pool.

Glucose primarily stimulates beta-cells through the activation of energy metabolism, required for the initiation of calcium signaling. As a secondary consequence, a surprising large number of signal transduction mechanisms are modulated during glucose stimulation. Such intracellular signaling is necessary to control the coordinated and time-dependent regulation of the above-mentioned biological processes. A main regulatory mechanism is reversible phosphorylation. In the beta-cell, several kinases and phosphatases have been demonstrated to influence glucose stimulated insulin secretion [18–20]. Key regulators include protein kinase A (PKA; [21]), protein kinase C (PKC; [22]), the insulin receptor and down-stream signaling kinases (reviewed in [23]), AMP activated protein kinase [24], CaMKII [25] and calcineurin [26] among others. Signaling via these kinases and phosphatases may also directly contribute to the regulation of metabolism-secretion coupling for instance at the level of mitochondria. In support of this hypothesis, a proteomic study has identified a number of mitochondrial phosphoproteins in insulin secreting cells [27]. Kinase-substrate relationship analysis revealed enrichment in PKC substrates. The data suggests kinases of the PKC family are upstream regulators of mitochondrial function. Consistent with these findings, we recently demonstrated that short-term regulation of PKC rapidly modulates glucose-stimulated mitochondrial respiration [28]. For a number of other signal transduction pathways, their role in glucose-induced respiration has not been investigated to date.

Several proteomic studies have identified phosphoproteins regulated during glucose-mediated beta-cell activation [29–33]. These studies have restricted themselves either to the study of a single time-point or have followed phosphorylation dynamics at very early stages of glucose-induced beta-cell activation. However, in response to a meal, glucose remains elevated for extended periods of time [1]. Here we have therefore compared regulation of protein phosphorylation from short-term (5 min) to later stages (30 and 60 min) of glucose stimulation. By analyzing dynamic changes of protein phosphorylation up to an hour, we may be able to gain insight into different phases of beta-cell activation during a meal. Furthermore, a main aim of this work was to determine whether there is a link between kinase-mediated signal transduction and mitochondrial activation beyond mitochondrial calcium signaling. Although the initial respiratory response is rapid, full activation of mitochondria by glucose requires up to one hour. Analyzing the dynamic changes in protein phosphorylation over this time-course may give us new insight into beta-cells signal transduction in relation to mitochondrial activation.

The here identified phosphoproteins were assigned to different biological processes regulated by glucose. Time-dependent phosphorylation of effector proteins gave us insight into kinetics of glucose-induced signaling and the coordinated regulation of protein translation, cytoskeletal rearrangements, ion homeostasis, insulin biogenesis, vesicle trafficking and insulin granule exocytosis. In addition, we used Kinase Substrate Enrichment Analysis (KSEA) to identify kinases and phosphatases responsible for the observed glucose-induced global phosphorylation changes. Respiratory analysis of glucose-stimulated insulin secreting cells (INS1) combined with pharmacological modulation of the KSEA regulated kinases demonstrated that a number of glucose regulated kinases impact on energy metabolism.

## Methods

### Reagents

Chemicals were from Sigma (Switzerland), Invitrogen (Switzerland), VWR (Switzerland) or Tocris (Switzerland) unless otherwise indicated.

### INS-1E cell culture

INS-1E cells were cultured at 37 °C in a humidified atmosphere (5% CO2) in RPMI-1640 medium (Invitrogen) containing 11 mM glucose, supplemented with 10 mM HEPES (pH 7.3), 10% (*v*/v) heat-inactivated fetal calf serum (FCS; Brunschwig AG, Switzerland), 1 mM sodium pyruvate, 50 μM β-mercaptoethanol, 50 μg/ml penicillin and 100 μg/ml streptomycin (Sigma, Switzerland).

### Mitochondrial respiration measurements

Oxygen consumption was measured using a XF96 instrument (Seahorse Biosciences, MA, USA). INS-1E cells were seeded into Seahorse tissue culture plates (Seahorse XF96 V3 PS Cell Culture Microplates #101085–004) at a density of 40,000 cells per well. 48 h later cells were washed twice and incubated in basal Krebs-Ringer bicarbonate HEPES (KRBH) buffer containing 2.5 mM glucose, 140 mM NaCl, 3.6 mM KCl, 0.5 mM NaH2PO4, 0.5 mM MgSO4, 1.5 mM CaCl2, 10 mM HEPES, and 5 mM NaHCO3 (pH 7.4) for 30 min at 37 °C inside the Seahorse instrument. Respiration rates were determined every 6 min at 37 °C using the following protocol 3 min of mixing were followed by 3 min of oxygen consumption measurements.

### Western-blots

Whole cells were lysed for 15 min on ice in RIPA buffer supplemented with protease inhibitors (Roche Applied Science, Switzerland), phosSTOP phosphatase inhibitor cocktail (Roche, Switzerland), 10 mM NaF, 0.1 μM PMSF and 2 mM Na-orthovanadate (Sigma, Switzerland) for complete phosphatase inhibition. The lysate was centrifuged at 14,000×*g* for 20 min at 4 °C, and the protein content of the supernatant was determined using the Pierce® BCA Protein Assay Kit (ThermoFisher, Switzerland). An amount of 25 μg of total protein was loaded on SDS-PAGE gels (Bio-Rad). For immunoblotting, proteins were transferred onto nitrocellulose membrane with i-blot (Invitrogene, Switzerland) and probed with the following antibodies: anti-pMARCKS-Ser167/170 (Cell Signaling #8722) anti-MARCKS (Cell Signalling #7756), anti-ERK (Cell Signalling #9102), anti-pERK-Thr202/Tyr204 (Cell Signalling #MA3–919), anti-tubulin (Chemicon #05–829), anti-pAMPK-Thr172 (Cell Signalling #2535), anti-AMPK (Cell Signalling #5831), anti-pACC-Ser79 (Cell Signalling #3661), anti-ACC (Cell Signalling #11818), anti-pAKT-Thr308 (Cell Signalling #2965), anti-AKT (Cell Signalling #9272), anti-pCREBS-Ser133 (Cell Signalling #9198), anti-CREBS (Cell Signalling #9197). Horseradish peroxidase-conjugated secondary antibodies were used followed by chemiluminescence detection (Amersham Biosciences, Switzerland).

### Phosphoproteomics and sample preparation

60 mm diameter petri dishes where seeded with 2 × 10^6^ INS-1E cells, and maintained in the incubator for 48 h until they reached 70–80% confluence. The day of the experiment, INS-1E cells were equilibrated at 37 °C in KRBH containing 2.5 mM glucose for 30 min. The plates were divided in two experimental groups and incubated either with 16.7 mM (high glucose) or maintained in 2.5 mM glucose in the same KRBH (low glucose). Subsequently, cell lysis was carried out after 5, 30 and 60 min on both groups. Lysates were prepared in RIPA buffer containing broad spectrum kinase and phosphatase inhibitors (Roche) at 4 °C. Protein concentrations were determined using the Pierce® BCA Protein Assay Kit. Following randomization of the samples and conditions (Additional file 1: Figure S1), samples containing 150 μg of proteins were taken for proteomic analysis and prepared in a final volume of 150 μl in 100 mM triethylammonium hydrogen carbonate buffer pH 8.5. Protein disulfide bridges were reduced with 10 mM tris(2-carboxyethyl)phosphine hydrochloride for 1 h at 55 °C. Alkylation was performed with 17 mM iodoacetamide for 30 min at room temperature in the dark. To remove lipids and salts, proteins were precipitated using methanol/chloroform. Methanol (400 μl), chloroform (100 μl) and H_2_O (300 μl) were added sequentially. Mixtures were centrifuged at 13,000 rpm (~ 18,500×g) for 5 min at 4 °C. Upper and lower phases were discarded. The white precipitates were washed with methanol (300 μl) and dried for 5 min. Protein pellets were suspended in 150 μl of 100 mM triethylammonium hydrogen carbonate buffer pH 8.5 and digested with an enzyme cocktail of trypsin/LysC (Promega, WI, USA) (1:50 *w*/*w*) at 37 °C overnight. The resulting peptides were isobarically labelled with tandem mass tags (TMT10plex™ from Thermo Scientific, IL, USA) by addition of 1.6 mg of TMT reagent in 82 μl of CH_3_CN for 1 h. The differentially labelled samples were pooled after reaction quenching with hydroxylamine. Samples were cleaned up using Oasis HLB cartridges (Waters, MA, USA), conditioning buffer (H_2_O/CH_3_CN/trifluoroacetic acid - TFA 5/94.9/0.1), loading/washing buffer (H_2_O/CH_3_CN/TFA 94.9/5/0.1) and elution buffer (H_2_O/CH_3_CN/TFA 49.9/50/0.1) as previously described^doi:^ 10.1007/978-1-4939-7057-5 and finally dried (an amount of ~ 500 μg (non-enriched fractions) for each TMT experiment (Additional file 1: Figure S1) was kept for reversed-phase liquid chromatography tandem mass spectrometry (RP-LC MS/MS) analysis). From 500 μg samples, isobarically-10plex phosphorylated peptides were enriched with TiO_2_ Mag Sepharose magnetic beads (GE Healthcare, Switzerland) following manufacturer instructions for enrichment of phosphopeptides (phospho-enriched fractions).

Samples (phospho-enriched and non-enriched fractions) were dissolved in H_2_O/CH_3_CN/formic acid 96.9/3/0.1. RP-LC MS/MS was performed on a hybrid linear ion trap-Orbitrap (LTQ-OT) Elite equipped with an Ultimate 3000 RSLC nano system (Thermo Scientific). Proteolytic peptides were trapped on an Acclaim PepMap 75 μm × 2 cm (C18, 3 μm, 100 Å) pre-column and separated on an Acclaim PepMap RSLC 75 μm × 50 cm (C18, 2 μm, 100 Å) column (Thermo Scientific) coupled to a stainless steel nanobore emitter (40 mm, OD 1/32″) mounted on a Nanospray Flex Ion Source (Thermo Scientific). The analytical separation of the phospho-enriched fractions was run for 150 min using a gradient that reached 30% of CH_3_CN after 140 min and 80% of CH_3_CN after 150 min at a flow rate of 220 nl/min. The analytical separation of the non-enriched fractions was run for 330 min using a gradient that reached 30% of CH_3_CN after 320 min and 80% of CH_3_CN after 330 min at a flow rate of 220 nl/min. For MS survey scans, the OT resolution was 120,000 at *m/z* = 400 (ion population of 1 × 10^6^) with an *m/z* window from 300 to 1500. For MS/MS with higher-energy collisional dissociation at 35% of the normalized collision energy and detection in the OT, ion population was set to 1 × 10^5^ (isolation width of 2 *m/z*), with resolution of 30,000 at *m/z* = 400, first mass at *m/z* = 100, and a maximum injection time of 250 ms. A maximum of 10 precursor ions (most intense) were selected for MS/MS. Dynamic exclusion was set for 60 s within a ± 5 ppm window. A lock mass of *m/z* = 445.1200 was used. Each sample was analyzed in triplicate.

Protein identification was performed using Mascot 2.4.0 (Matrix Sciences, UK) against the UniProtKB rat proteome database (27/08/2014 release; 28,900 entries). Trypsin was selected as the proteolytic enzyme, with a maximum of 2 potential missed cleavages. Peptide and fragment ion tolerance were set to, respectively, 10 ppm and 0.02 Da. Mascot result files from both phospho-enriched and non-enriched fractions were loaded into Scaffold Q + S 4.3.2 (Proteome Software, OR, USA) for sample normalization purposes and further searched with X! Tandem (The GPM, thegpm.org; version CYCLONE (2010.12.01.1)). For the phospho-enriched fractions, we considered carbamidomethylation of Cys, TMT-labeling of Lys and TMT-labeling of peptide amino termini as fixed amino acid modifications, and oxidation of Met, deamidation of Asn and Gln, and phosphorylation of Ser, Thr and Tyr as variable modifications. For the non-enriched fractions, we considered carbamidomethylation of Cys as fixed amino acid modifications, and oxidation of Met, deamidation of Asn and Gln, acetylation of Lys, TMT-labeling of Lys and peptide amino termini, and phosphorylation of Ser, Thr and Tyr as variable modifications. Based on a target-decoy strategy, both peptide and protein false discovery rates were fixed at 1% maximum, with a one-unique-peptide criterion to report protein identification. Scaffold PTM 3.0.0 (Proteome Software) was used to annotate post-translational modifications (PTMs) from Scaffold results. Using the site localization algorithm developed by Beausoleil et al., doi:10.1038/nbt1240 Scaffold PTM re-analyzes tandem mass spectra identified as modified peptides and calculates Ascore values and site localization probabilities to assess the level of confidence in each PTM localization. Scaffold PTM then combines localization probabilities for all peptides containing each identified PTM site to obtain the best-estimated probability that a PTM is present at that particular site. Minimum localization probability was set to 95%. Isobaric tagging quantitative values for phosphorylated peptides were exported from Scaffold PTM and relative quantification of phosphorylation sites was computed using an in-house script with R version 3.1.1 (http://www.r-project.org/).

Paired *t*-test was applied to identify proteins and p-sites displaying *p*-values≤0.05 between the low glucose and high glucose groups at every time point. Regulation threshold was fixed at 23%, i.e., Log2 fold change (FC) > 0.3 or < − 0.3.

### Gene ontology enrichment, heatmap generation and identification of trajectory clusters

Significantly regulated phosphoproteins were assigned to gene ontology (GO) categories for cellular processes and cellular localization using Metacore (GeneGo, MI, USA). The *p*-values for networks and processes were all calculated using the same basic formula for hypergeometric distribution. The *p*-value essentially represents the probability for a particular mapping of an experiment to network or process to arise by chance, considering the numbers of genes observed in the experiment versus the number of genes in the network or process within the “full set” of genes in these networks or processes. The statistical programming language R and its packages ggplot, cluster and RColorBrewer were used to plot the heatmaps. For the ordering of the variables on the heatmaps, hierarchical clustering was performed on the phosphorylation sites using the following parameters: complete linkage and Gower as the metric used for obtaining the pairwise dissimilarity. The function ‘daisy’ from the R package ‘cluster’ was used to obtain the distances between variables. Gower’s distance, which allows missing values, computes distances between 2 data points as the sum of all the variable-specific distances. When all the variables are quantitative, like the phosphorylation sites analyzed, the distance is obtained using the more standard Manhattan metric. The code to draw the heatmaps was an adaptation from the original ggpheatmap function written by Chris Wallace (University of Cambridge, UK).

The traj R package for clustering longitudinal trajectories was used to identify the clusters of proteins (using 1-norm distances [34], k = 4 and discarding the 19th measure, i.e., the mean of the second differences). The hierarchical ordering of the GO enrichment heatmaps was obtained using the R hclust routine.

### Kinase substrate enrichment analysis (KSEA)

KSEA was performed as described in by Casado et al. [35]. PhosphositePlus and Signor Databases (https://signor.uniroma2.it/) were used as the kinase-substrate relationship dataset resource. Essentially, phosphosites identified at 5, 30 and 60 min upon glucose stimulation were arranged into substrate groups defined as containing phosphorylation sites known to be substrates of specific kinases across kinase-substrate relation databases or as sharing specific phosphorylation motifs. Then, we calculated the extent and statistical significance of the enrichment of these substrate groups relative to the particular phosphoproteomic data set.

## Results

### Time-dependent protein phosphorylation during glucose stimulation of INS-1E cells

**Fig. 1:**
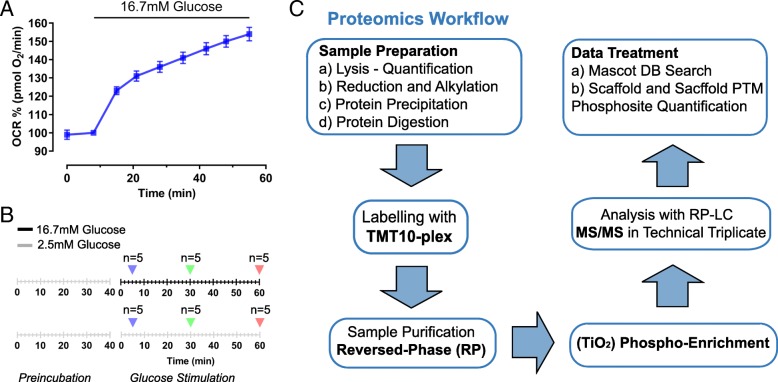
Time course phosphoproteomics during glucose stimulation in INS-1E pancreatic beta-cells. **a** Effect of glucose stimulation on mitochondrial respiration of INS-1E pancreatic β-cells stimulated. **b** Experimental design to identify short-term glucose dependent regulated phosphoproteins over time. **c** Proteomic workflow for the digestion, enrichment, identification and quantification of phosphopeptides from INS-1E protein lysates

**Fig. 2:**
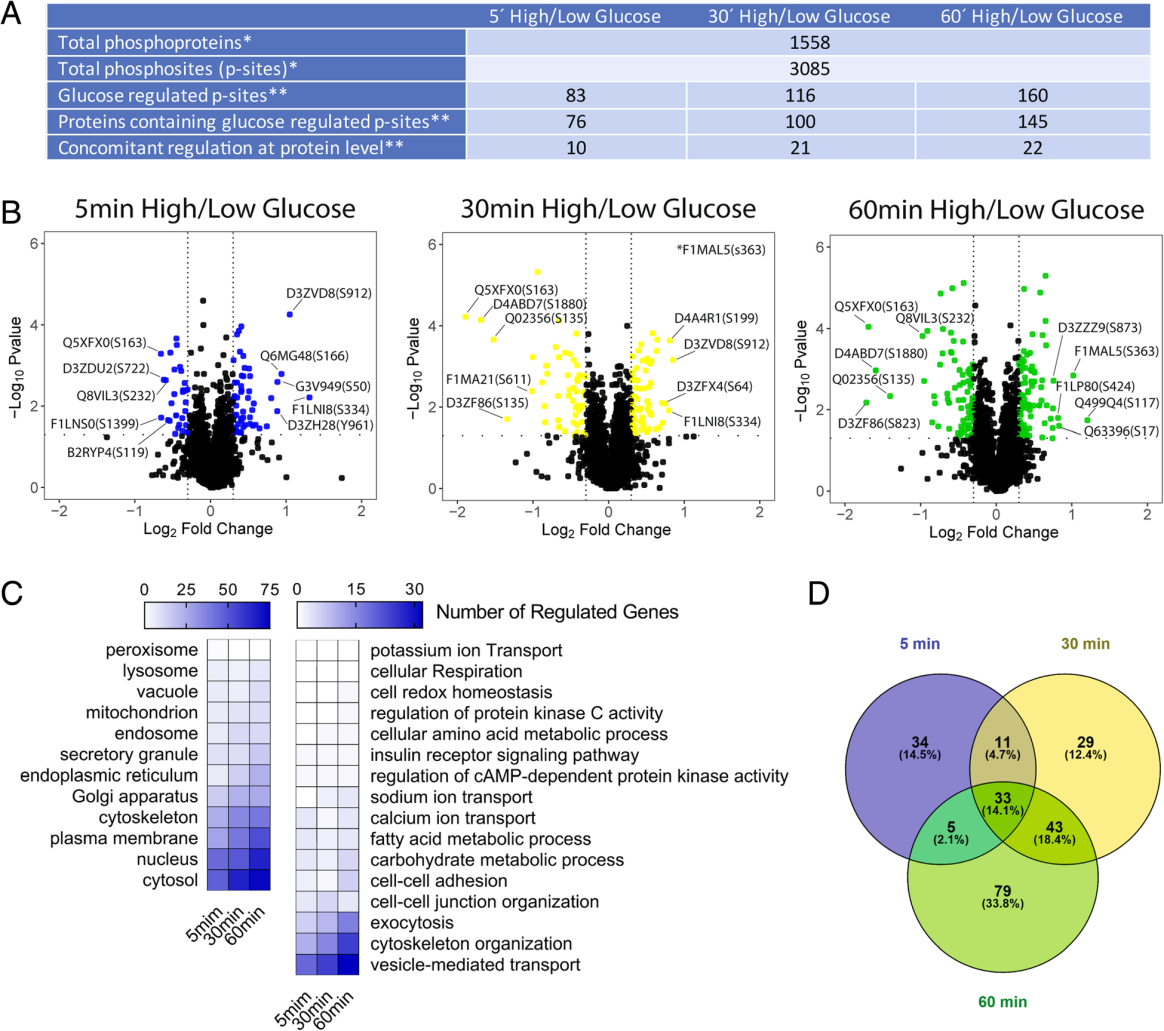
Glucose-dependent regulated phosphoproteins and phosphorylated sites (p-sites) overtime. **a** Table (*successfully quantified at least twice in all experimental conditions; **p-value < 0.05 and FC (Log2) > 0.3 or < − 0.3). **b** Volcano plots displaying the distribution of significant regulated p-sites overtime. P-sites significantly changed (p-value < 0.05) and FC (Log2) > 0.3 or < − 0.3 compared to control are shown in color. **c** Heat maps, showing enrichment of cell process related with insulin secretion, and cell localization / organelle enrichment. **d** Venn diagrams showing numbers and percentages of common and differentially regulated p-sites overtime

Nutrient activation of pancreatic beta-cells occurs in several time-dependent steps. At the level of mitochondrial respiration, the process has been dissected in an early calcium independent (first few minutes) and a second long lasting activation (> 30 min) (Fig. 1a; [11]). As these metabolic changes occur, glucose stimulation also initiates a number of signal transduction pathways, which are important for the regulation of insulin secretion in pancreatic beta-cells [19]. Here we have analyzed the kinetic changes of protein phosphorylation during glucose stimulation of INS-1E cells in order to improve our understanding of glucose-induced signal transduction and its possible link to mitochondrial activation. We followed protein phosphorylation over time, using MS-based phosphoproteomics. INS-1E cells were stimulated with glucose (16.7 mM) for 5, 30 and 60 min (Fig. 1b) corresponding to different phases of the beta-cell respiratory response (Fig. 1a). For each time-point, control cells maintained under resting glucose conditions (2.5 mM glucose) were also extracted (Fig. 1b). A proteomic workflow for the digestion, enrichment, identification and quantification of phosphopeptides from INS-1E protein lysates was used (Fig. 1c). This experimental design allows the identification of glucose-dependent changes over time excluding confounding phosphorylation events not primarily dependent on glucose activation, such as phosphorylation changes occurring during incubation in KRBH medium. Protein lysates from each of the three high glucose and matching control conditions were prepared in five independent experiments and the derived phosphopeptides quantified with MS. This strategy led to the identification of 3085 unique phosphorylation sites (p-sites) present in 1558 proteins (≥ 99% certainty of p-site localization; Fig. 2a). The distribution of p-sites per proteins is shown in Additional file 2: Figure S2. Only peptides present in all experimental conditions and at least two out of five replicates per condition were included in the quantitative analysis. After 5, 30 and 60 min of glucose stimulation the phosphorylation status of 83, 116, and 160 p-sites were respectively altered when compared to INS-1E cells maintained in resting glucose (2.5 mM) (Fig. 2a and Additional file 3: Table S1, Additional file 4: Table S2 and Additional file 5: Table S3). The data for the three different time points were expressed as volcano plots comparing fold-changes of glucose-induced phosphorylation versus the statistical significance of the glucose-induced changes (Fig. 2b).

Given the period of stimulation, synthesis or degradation of proteins cannot be excluded [36]. To asses this possibility, we also interrogate our samples at the proteome level (Additional file 6: Figure S3A). The study revealed that only a small fraction of the p-sites regulated proteins also undergo significant changes at the total protein level following glucose stimulation, 13% (10/76), 21% (21/100) and 15% (22/145) respectively at 5, 30 and 60 min (Fig. 2a, Additional file 6: Figure S3B and Additional file 7: Table S4). Moreover, when common, the extent of the regulation at the protein level was often significantly lower, as visualized by the vertical spread of the points in the p-site versus protein regulation correlation plots (Additional file 6: Figure S3C).

Phosphorylation patterns at first sight seem similar between the different time-points with about equal number of p-sites with increased and decreased phosphorylation status. However, the total number of p-sites increased over time (Fig. 2a). When looking at the p-sites regulated by phosphorylation, only a small fraction was significantly changed over the entire time-course of glucose stimulation compared to control. The Venn diagram (Fig. 2d) shows the distribution of significantly regulated p-sites at the different time points and the number of changes shared between two or threesampling conditions. Among the p-sites significantly changed following 5 min of glucose stimulation 41% (from 83 p-sites) were exclusively regulated at this early time-point. When comparing 30 and 60 min of glucose stimulation, the overlap of significantly glucose regulated phosphorylation events was larger but still small. Overall, only 33 p-sites (14.1% of the total regulated p-sites) were changed by glucose at all the time points following initiation of the nutrient response (Fig. 2d). These findings emphasize the importance to look into the temporal aspects of glucose-dependent signal transduction in insulin secreting cells.

GO localization enrichment analysis revealed that the majority of p-sites undergoing significant glucose-induced changes were found in cytoplasmic, nuclear, plasma membrane, cytoskeletal and Golgi proteins (Fig. 2c and Additional file 8: Table S5). In other compartments, few or no p-sites were regulated by glucose. We also calculate enrichment of proteins involved in cellular processes described in the literature for having an important role in short-term regulation of nutrient-induced insulin secretion (Fig. 2c and Additional file 9: Table S6). In our data set, p-sites significantly regulated by glucose were enriched in cytoskeletal proteins, regulators of the cytoskeleton, cell-cell junction organization and proteins involved in vesicle-mediated transport as well as exocytosis (Fig. 2c). Phosphorylation of proteins reflecting these processes were altered with time following glucose stimulation.

Time point-specific regulated p-sites (5 min: 34, 30 min: 29, and 60 min: 79; Fig. 2d) may reveal further differences on cell functions/pathways differentially regulated at the phosphorylation level over time. To identify differentially affected GO categories between groups, the standard deviation of the –Log10 (*p*-value) amongst the three groups was calculated for every GO term and then the whole ontology was sorted in decrease order of standard deviation (Additional file 10: Table S7). Additional file 11: Figure S4 displays the top 30 GO terms of such list. These results revealed that a number of phospho-proteins exclusively regulated at 5 min are responsible of nuclear mRNA processing and chromatin disassembly. On the other hand, a number of phospho-proteins only regulated at 60 min were involved in the regulation of actin organization dynamics as well as mRNA processing.

### Identification of p-site clusters with similar phosphorylation kinetics

Our GO cell process enrichment (Fig. 2c and Additional file 8: Table S5) points to the regulation of multiple cellular processes, linked to different degrees with glucose-dependent beta-cell activation. In order to capture phosphorylation kinetics across our full dataset, we clustered phosphorylation events that displayed similar changes over time. A 3-step procedure to identify clustering of longitudinal trajectories was applied [34] (see also Materials and Methods). This method calculates different measures, which describe trajectories’ features, selects the most informative measures by performing a factor analysis, and clusters the data points based on these selected factors. This method has been used successfully to cluster longitudinal trajectories during disease progression [34, 37].

**Fig. 3:**
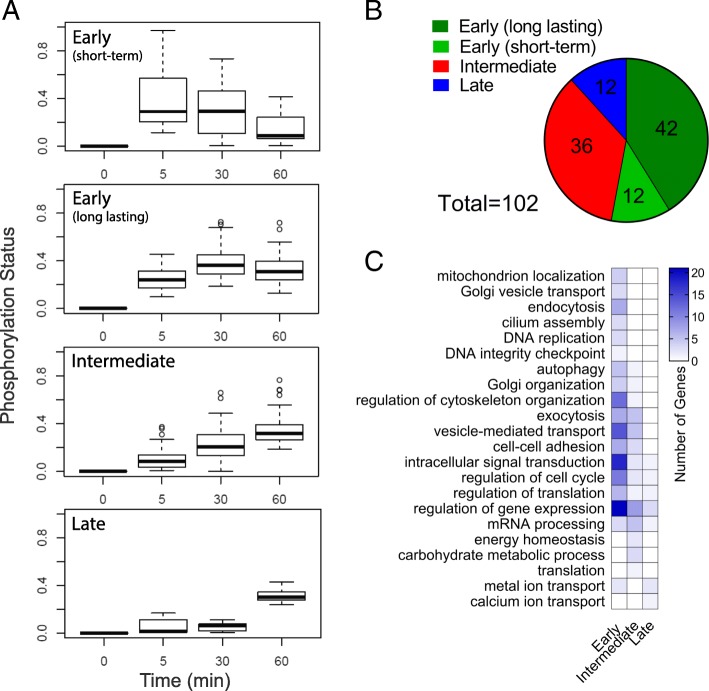
Systematic identification and clustering of glucose-dependent dynamic phosphorylation profiles. **a** Analysis of p-site trajectories identified four main clusters according to its phosphorylation dynamics overtime: early (short-term), early (long-lasting), intermediate and late regulated. The p-site dataset included in the analysis of trajectories was formed by the 234 p-sites matching the *p*-value < 0.05 and FC (Log2) > 0.3 or < − 0.3 criteria in Fig. 2d. **b** P-site status distribution on the four clusters identified. **c** Heat-map showing distribution of glucose phospho-regulated cell processes across different clusters

Based on this analysis, we separated the 102 most markedly changed p-sites into four distinctive clusters (Fig. 3a). The first two clusters contain phosphorylation events rapidly changing in response to nutrient stimulation (within 5 min; Fig. 3a, b). P-sites reflecting such rapid changes in phosphorylation status can be further subdivided in long-lasting changes in phosphorylation (42 p-sites, cluster 1) and early transient phosphorylation (12 p-sites, cluster 2). The other two clusters comprise p-sites whose phosphorylation status changes after 30 min (Fig. 3a, b; intermediate; 36 p-sites, cluster 3) or only after 60 min (Fig. 3a, b; late; 12 p-sites, cluster 4). The results highlighted the generally fast activation of signal transduction in response to glucose.

Clustering of time-resolved p-site trajectories can uncover functionally linked proteins and cellular process [38]. To this end, members of the different clusters were assigned to GO and displayed in an unsupervised heatmap (Fig. 3c and Additional file 12: Table S8). The results show an early coordinated phospho-regulation of elements linked to signal transduction, cytoskeleton organization, gene expression, mRNA processing and stability, vesicle trafficking, vesicle-mediated exocytosis and cell-cell adhesion. Phosphorylation of proteins associated with energy homeostasis, carbohydrate metabolism and calcium ion transport was delayed (Fig. 3c).

### Phosphorylation of proteins involved in biological processes directly linked to beta-cell activation

**Fig. 4:**
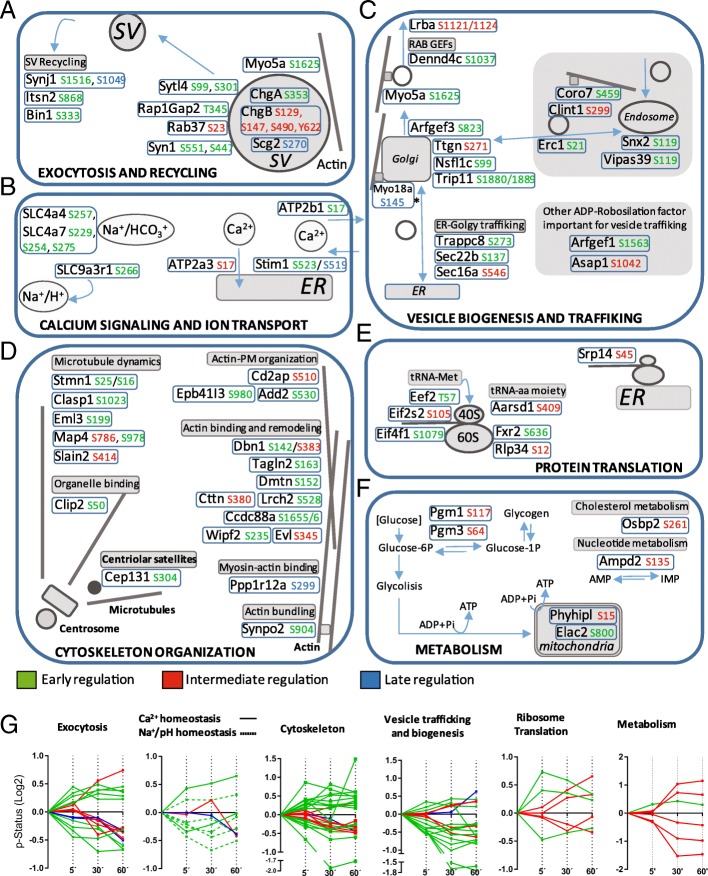
Time-course of significantly regulated p-sites in the context of cell process important for short-term regulation of glucose-induced insulin secretion. Panels includes phospho-regulated proteins in the context of, exocytosis (**a**), calcium and ion homeostasis (**b**), vesicle trafficking and biogenesis (**c**), cytoskeleton organization (**d**), protein translation (**e**) and metabolism (**f**). Fast glucose-regulated p-sites are colored in green, late regulated p-sites are colored in blue and p-sites displaying an intermediate regulatory-kinetic are colored in red. **g** P-site kinetics of proteins involved in the above mentioned cell process. P-sites included undergoes more than FC (Log2) > 0.3 or < − 0.3 regulation during at least some of the three time points studied (5, 30 and 60 min) and *p*-value ≤0.05

Glucose-dependent insulin secretion engages a large number of biological processes directly involved in the full activation of beta-cells. Proteins whose phosphorylation status was strongly regulated (*p*-value< 0.05 andFC (Log2) > 0.3 or < − 0.3) over the time-course were assigned to such biological processes including insulin granule exocytosis and membrane recycling (Fig. 4a), calcium signaling and pH homeostasis (Fig. 4b), vesicle biogenesis and trafficking (Fig. 4c), actin and tubulin cytoskeleton dynamics (Fig. 4d), protein synthesis (Fig. 4e) and metabolism (Fig. 4f).

Consistent with the rapid activation of exocytosis, calcium signaling, cytoskeleton dynamics and vesicle trafficking, most proteins in these categories were significantly regulated already after 5 min, the shortest time-point assessed after initiation of the glucose stimulus (Fig. 4g). Both rapid (5 min) and delayed regulation (30 min) was measured for proteins controlling protein translation. The few phosphorylated metabolic enzymes were mostly regulated at the later two time-points (30 and 60 min; Fig. 4g).

While it is impossible to describe all the phosphoproteins regulated during short-term glucose stimulation, we highlight a few of them in each functional group. Rapid phospho-regulation was noted in several proteins mediating and controlling the final steps of insulin granule transport and exocytosis (Myo5a, Sytl4, RapGap2, Rab37 and Syn1). Synaptotagmin-like protein 4/Granuphilin (Sytl4) is particularly interesting, given its well-described role in insulin granule exocytosis through its interaction with the Rab3 GTPase and the exocytosis regulator Munc-18/Sec1 [39]. Several proteins in Fig. 4a suggest that clathrin dependent recycling from the plasma membrane may be regulated by rapid phosphorylation (Synj1, Itsn2 and Bin1). Furthermore, three chromogranins (ChgA, ChgB and Scg2) involved in granule biogenesis and insulin exocytosis [6, 40] were dynamically phosphorylated upon glucose stimulation in INS-1E cells. Phosphorylation of these proteins might points to the existence of a still elusive glucose regulated kinase localized in the lumen of secretory granules [41].

Proteins controlling cytosolic pH (SLC4a4, SLC4a7, SLCa3r1) were also rapidly regulated (Fig. 4b). SLC4A4 and 7 are plasma membrane Na^+^/HCO_3_^−^ exchangers expressed in pancreatic beta-cells [42], whereas SLC9a3r1 encodes NHERF1 a regulatory cofactor of the Na^+^/H^+^ exchanger, important to maintain cellular pH homeostasis.

Three key regulators of cytosolic Ca^2+^ fluxes were also phospho-regulated in our dataset: the plasma membrane Ca^2+^-ATPase (PMCAs _Ser17_; ATP2b1), the sarco-endoplasmic reticulum Ca^2+^-ATPase (SERCAs _Ser17_; ATP2a3), and the stromal interaction molecule 1 (Stim1 _Ser519/Ser523_), a key component of store operated Ca^2+^ entry (SOCE) [43] (Fig. 4b). The two p-sites on Stim1 _Ser519/523_ are located in the C-terminal domain of the protein, interacting with Orai1 at the plasma membrane. Alternative regulated p-sites of Stim1 _Ser486/575/608/631/688_ have been found previously in the cytosolic tail of the protein between the CAD domain and the C-terminal amino acid. Phosphorylation strongly modulate Stim1/Orai1 interaction, required for the regulation of store operated Ca^2+^ fluxes [44, 45]. A number of studies point to a role for SOCE in the control of Ca^2+^ homeostasis/signaling during beta-cell activation mediated by nutrients [43, 46]. We provide additional evidence that the temporal control of phospho-regulation in the C-terminal tail of Stim1 is an important mechanism for the temporal control of SOCE during glucose-induced insulin secretion.

Several p-sites belonging to proteins engaged in vesicle trafficking and biogenesis were rapidly regulated by glucose (Fig. 4c). These proteins are essential components for vesicular traffic between the endoplasmic reticulum (ER) and the Golgi (Sec16a, Sec22b and Trappc8), control of Golgi function and morphology (Ttgn, Nsfl1c, Trip11, Myo18a and Arfgef1), insulin granule biogenesis (Arfgef3), granule transport and polarized secretion (Lrba, Dennd4c and Myo5a) and the bidirectional vesicular transport between the Golgi and endosomes (Coro7, Clint1, Erc1, Snx2, Vipas39). The fact that Sec16 which defines ER exit sites and Sec22b involved in the recycling of COPII coated vesicles undergo glucose-dependent phosphoregulation indicates that already very early steps in insulin trafficking are likely controlled by glucose [47]. A very interesting protein is Arfgef3/BIG3. This Arf-GTP exchange factor localizes to the trans Golgi network where it acts as a negative regulator of insulin granule biogenesis [48]. Glucose-dependent phosphorylation may be a mechanism to inhibit ArfGef3 thereby enhancing the number of newly formed granules. The actin based motor protein Myo5a is another interesting phosphoprotein. It mediates the transport of insulin granules to the plasma membrane and binds the Rab-interacting protein MyRIP. cAMP dependent phosphorylation of MyRIP promotes interactions with the Rab protein rabphilin-3A and Myo5a. Whether Myo5a phosphorylation is contributing to the regulation of granule transport remains to be investigated.

Both the tubulin and actin cytoskeleton play an important role in the efficient delivery of secretory granules to docking sites at the plasma membrane [49]. Phosphorylation of a large number of proteins regulating tubulin (Stmn1, Clasp1, Eml3, Map4 and Slain2) and actin (Cd2ap, Ebp41I3, Add2, Dbn1, Tagln2, Dmtn, Cttn, Lrch2, Ccdc88a, Wipf2, Evl, Ppp1r12a and Synpo2) dynamics and structure was observed (Fig. 4d). The majority of these phosphorylation changes occur within the first 5 min of glucose stimulation. Stmn1 (Stathmin1) regulates the tubulin cytoskeleton by destabilizing microtubule filaments. Glucose caused phosphorylation changes on S16 and S25 of Stmn1 (Fig. 4d). Phosphorylation of S16 is known to regulate the ability of Stmn1 to control microtubule polymerization [50]. In the context of the beta-cell it is interesting to note that Stmn1 expression is upregulated during pregnancy-induced islet expansion [51]. Wipf2 (WAS/WASL-interacting protein family member 2) may be an important regulator of the actin cytoskeleton [52]. Wipf2 binds to N-WASP regulating actin dynamics close to the plasma membrane. N-WASP in turn through the regulation of the Arp2/3 complex controls second phase insulin secretion [17].

Glucose-induced insulin secretion is also closely associated with a pronounced increase in protein translation and insulin biosynthesis [13, 14]. Aligned with these findings, we observe regulation by phosphorylation of proteins required for protein translation and targeting of the protein nascent chain to the ER (Eef2, Eif2s2, Eif4f1, Aarsd1, Fxr2, Rlp34 and Srp14; Fig. 4e). We find two translation initiation factors (Eif2s2, Eif4f1) controlled by phosphorylation. These changes similar to dephosphorylation of the translation initiation factor 2α by protein phosphatase 1 [14] may contribute to glucose-induced activation of protein biosynthesis. Similarly, protein elongation may be controlled by phosphorylation of Eef2 on T57 (Fig. 4e). Indeed, when T57 is phosphorylated Eeef2 activity is inhibited [53]. Glucose-induced dephosphorylation of this protein may therefore accelerate protein elongation.

We could only find a few metabolic enzymes regulated by phosphorylation (Pgm1, Pgm3, Osbp2, Ampd2, Phyhipl and Elac2; Fig. 4f), which for most part were differentially phosphorylated only after 30–60 min (Fig. 4g). Although glycolysis and mitochondrial metabolism of glucose-derived pyruvate are almost immediately accelerated following glucose stimulation, these kinetic changes are not likely influenced by regulation of enzyme activity by phosphorylation. An interesting phosphoprotein in this group is phosphoglucomutase Pgm1. The enzyme converts glucose 1-phosphate from glycogenolysis to the glycolytic intermediate glucose 6-phosphate and vice versa. S117 of Pgm1 is phosphorylated as part of the catalytic mechanism of this reaction [54]. Augmented S117 phosphorylation therefore likely reflects active conversion of glucose 6-phosphate to glucose 1-phosphate for glycogen synthesis in the continued presence of elevated glucose.

In summary, this overview of glucose-regulated phosphorylation highlights some of the complexity how signal transduction regulates beta-cell functions. We conclude that phosphorylation kinetics of proteins controlling early events in glucose-induced insulin secretion are rapid.

### Dynamic regulation of kinases and phosphatases by glucose

**Fig. 5:**
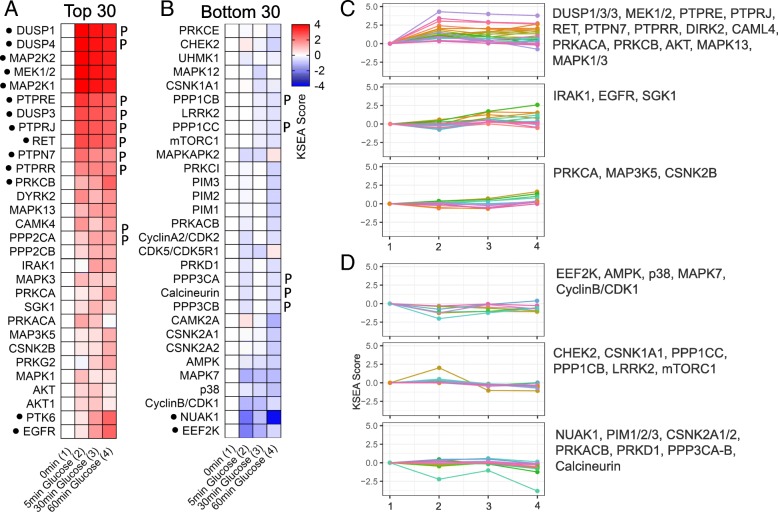
Kinase substrate enrichment analysis over a time-course. **a** Heatmap showing distribution of top 30 KSEA scores kinase and phosphatases (highlighted with “P”) activities after 5, 30 and 60 min of continuous glucose stimulation. **b** Heatmap showing distribution of bottom 30 KSEA scores kinase and phosphatase activities after 5, 30 and 60 min of continuous glucose stimulation. For kinases, high and low KSEA scores denote activation and inhibiton respectively; conversely for phosphatases, high and low KSEA scores denote inhibiton and activation respectively. Kinases and phosphatases displaying KSEA scores <− 2 or 2 < are highlighted with a black dot. **c**-**d** Trajectory clusters reporting temporal profiling of individual kinase/phosphatase activities. **c** Clusters undergoing positive KSEA scores upon glucose stimulation. **d** Clusters undergoing negative KSEA scores upon glucose stimulation

Starting from our kinetic phospho-proteomic datasets, we used KSEA [35]) to infer changes in kinase activities. Briefly, 125 out of 3086 identified p-sites were matched to Kinase-substrate relationship databases and KSEA scores were calculated for every experimental replicate. The output of the analysis provided evidence on the possible activity of 122 different kinases and phosphatases (Additional file 13: Figure S5**)**. Subsequently averages of replicates were calculated and hierarchical clustering was applied to display the 30 most significantly activated and inhibited kinases and phosphatases (Fig. 5a-b).

The results of KSEA showed that the protein kinases MAP2K1/2 (also known as MEK1/2) were significantly activated at all time points. Moreover the analysis revealed a trend of activation for DYRK2, a dual-specificity protein kinase, PRKCA and -B (Protein kinase C), MAPK13 (a member of p38 MAPK family), CAMK4, MAPK3 and-1 (ERK1/2), PRKACA (Protein kinase A) and AKT (Fig. 5a). Conversely, KSEA reported significant inhibition of EEF2K and NUAK and displayed inhibitory trends for AMPK, MAPK-p38, Cdk1/CyclinB and MAPK7 (Fig. 5b). Interestingly KSEA revealed significant inhibition of the non-receptor dual-specificity phosphatases DUSP-1, − 3 and − 4, the membrane receptors with tyrosine phosphatase activity PTPRE, PTPRJ, PTPRR and the non-receptor tyrosine phosphatase PTPN7 (Fig. 5a). In contrast, the analysis showed a slightly higher activity of Calcineurin (PPP3CA and PPP2CB) upon glucose stimulation (Fig. 5b).

For several well-known kinases such as Raf, PKC family, PKA, AKT, AMPK and Eef2k, we looked at the time-course of effector protein phosphorylation. Furthermore, we used p-site specific antibodies to follow phosphorylation changes on a specific substrate of each of these kinase families by Western blotting. Quantification of effector protein phosphorylation over time reveals the basis of KSEA prediction of kinase activity and the robustness of this approach (Additional file 14: Figure S6). Analysis of specific protein phosphorylation changes by Western blotting allowed us to obtain independent confirmation of the results. We observed rapid and robust phosphorylation of substrates of the Raf MAP kinase pathway in response to glucose (Additional file 14: Figure S6A) using both, MS-derived data and immuno-based results. Phosphorylation of the PKC (Additional file 14: Figure S6B), PKA (Additional file 14: Figure S6C) and Akt (Additional file 14: Figure S6D) family substrates was equally rapid but less marked than expected from MS-derived data. MS-based phosphoproteomics and Western analysis also demonstrated rapid inactivation of AMPK (Additional file 14: Figure S6E) and Eef2k (Additional file 14: Figure S6F) activity.

Time resolved kinase/phosphatases activities can give new insights into the temporal regulation of glucose stimulated insulin secretion. To this end, we identified groups of kinases/phosphatases according to their temporal KSEA score trajectories. Overall, we note that when classifying the kinases/phosphatases by which direction they present their largest regulation (measured as changes in KSEA score over time during three consecutive time points) nearly 70% of the them are in the group of positive KSEA scores already at 5 min (Fig. 5c and Additional file 15: Figure S7). In contrast, for half of the nearly 30% kinases/phosphatases displaying negative KSEA scores, the reduction occurs only after 60 min (Fig. 5d and Additional file 15: Figure S7). Kinases and phosphatases belonging to the top 30 positive and negatively regulated enzymes assigned to specific trajectories are listed in Fig. 5c and d. Kinases and phosphatases regulated early on should influence both first and second phase insulin secretion. Those undergoing changes at intermediate or late time-points may primarily affect the second phase of the secretory response.

### Glucose-regulated kinases contributing to the regulation of mitochondrial energy metabolism

We hypothesized that glucose-induced signal transduction contributes to the full respiratory response beyond the role of calcium in the stimulation of beta-cell mitochondria. To test the relevance of different cytosolic signal transduction pathways predicted with KSEA, we used pharmacological inhibitors and activators and tested to what extent they affected glucose-induced respiration in INS-1E cells. A total of 27 compounds were tested at three different concentrations (Additional file 16: Table S9).

**Fig. 6:**
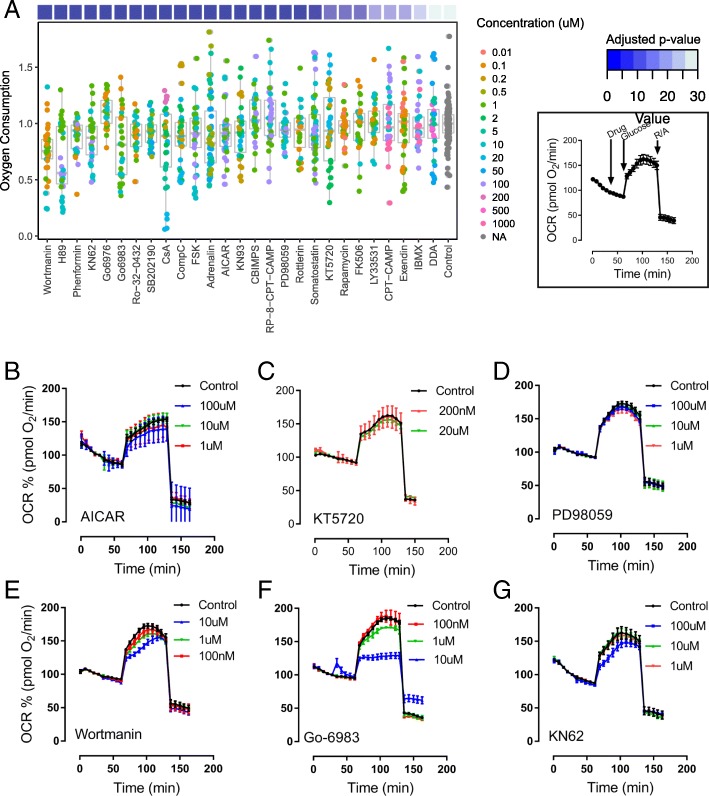
Effect of modulators of KSEA-identified kinases on glucose-stimulated mitochondrial respiration. **a** Normalized effect of drugs tested on glucose-stimulated mitochondrial respiration, including *p*-values. In the inset, oxygen consumption recording in INS-1E cells. Protocol designed to identify the effect of glucose-regulated kinases on glucose-driven mitochondrial respiration. From **b** to **g**) Effect of AICAR, PD98059, KT5720, Wortmanin, Go-6983 and KN62, on glucose-stimulated mitochondrial respiration

Respiration was measured in INS-1E cells kept initially at resting glucose (2.5 mM) concentrations. At this point, inhibitors and activators of specific signal transduction pathways were added to determine whether the pharmacological agents affected basal respiratory rates. Thirty minutes later, the INS-1E cells were stimulated with glucose to accelerate respiration (Fig. 6a inset). The respiratory responses to glucose (16.7 mM) in the presence of pharmacological agents were quantified as the area under the curve over basal respiration and compared to the glucose response in mock treated cells. An overview of the fold changes and statistical significance compared to the control respiratory response is shown in Fig. 6a. The large majority of compounds tested caused no significant changes in the respiratory response to glucose. Manipulation of PKA activity did not alter mitochondrial energy function. Agents raising or lowering cAMP and the PKA inhibitor KT5720 (see also Fig. 6c) did not affect the respiratory response. Only the PKA inhibitor H89 caused a highly significant reduction of the glucose-induced respiratory response (Fig. 6a), however H89 affected respiration in general even under resting glucose conditions (Additional file 17: Figure S8). PKA activity may affect mitochondrial respiration but stimulation of PKA is not required for the acute glucose stimulated respiratory response. Acute manipulation of AMPK does not influence respiration in INS-1E cells. Inhibition of AMPK with compound-C or activation of AMPK with AICAR (to prevent glucose-induced inhibition of AMPK) had no effect on glucose-induced respiration (see also Fig. 6b). The exception was the AMPK activator phenformin, which inhibited respiration in INS-1E cells (Additional file 17: Figure S8). Most likely reduction of respiration by phenformin happens via the inhibition of complex I of the respiratory chain rather than the activation of AMPK [55]. Although MEK signaling is strongly activated by glucose (Fig. 5a), this pathway does not appear to contribute to glucose-induced respiration. We observed no alteration of respiration when the MAP kinase pathway was blocked at the level of MEK1 using PD98059 (Fig. 6c).

On the other hand, our pharmacological data suggest that PI3Kinase, PKC and calmodulin-dependent kinase type-II may contribute to acute glucose-induced activation of mitochondrial function. Drugs targeting the above-mentioned kinases lowered the full respiratory response to glucose without significantly changing resting respiratory rates. The phosphatidylinositol 3-kinase inhibitor wortmannin (0.1–10 μM) reduced glucose-induced respiration in a dose dependent manner (Fig. 6e). Consistent with our earlier published results [28], PKC inhibition (Go6983) reduced glucose-induced respiration (Fig. 6f). Finally, KN-62, a calmodulin-dependent kinase type II inhibitor caused a significant reduction of respiratory rates during glucose activation of INS-1E cells (Fig. 6g).

## Discussion

Insulin secretion from the pancreatic beta-cell is biphasic. The first phase is dependent on a readily releasable pool of granules. Interestingly, during glucose clamp experiments, secretion during the second phase continuous to rise gradually for 2 h and more. Such findings suggest that full activation of beta-cells may occur over an extended period of time. Full activation of beta-cells may occur gradually to adjust to the energy load and duration of a meal. Similarly, glucose activation of beta-cell mitochondria is almost instantaneous but subsequently respiratory rates increase steadily for up to one hour in a calcium dependent manner.

The transition of beta-cells from a resting to an activated state goes beyond its metabolic activation by glucose and consequent promotion of insulin secretion. Glucose stimulation also affects a large number of biological processes linked to insulin biosynthesis, early membrane trafficking and insulin granule biogenesis and transport. Stimulation of these biological processes suggests the loss of insulin granules due to exocytosis needs to be balanced by de novo synthesis. To coordinate the chain of events from insulin biosynthesis to exocytosis, glucose indirectly activates a number of signal transduction pathways. It is interesting that glucose alone is able to initiate these signaling pathways without the assistance of peptide hormones and neurotransmitters known to potentiate insulin secretion. Stimulation of these signaling pathways may contribute to gradual beta-cell activation during the second phase of insulin secretion.

The initial response to glucose (5 min) coinciding with first phase insulin secretion was compared to increasing times of continuous glucose stimulation occurring during second phase insulin secretion (30 min) and later activation (60 min). We observed a total of 234 p-sites significantly regulated by glucose. The large majority of glucose dependent changes were limited to one or two time-points. Only 33 p-sites were significantly and commonly regulated at the three time points. Our findings as well as earlier phosphoproteomics studies [31–33] underline the importance of studying the kinetics of glucose-induced activation in insulin secreting cells. Many p-sites sites identified here in insulin secreting cells have been detected previously in large-scale proteomic surveys across multiple rat, mouse or human tissues [56, 57]. For example, this is the case for 50 of the 83 p-sites displayed in Fig. 4. Identification of specific p-sites in multiple tissues supports a possible regulatory role and by extension a regulatory role in beta-cells. In fact, a large number of p-sites have already been revealed by earlier beta cell phospho proteomic studies [31–33].

To properly describe the kinetics of phosphorylation during glucose activation, we applied a clustering method of p-site trajectories. We identified four groups of p-sites with distinctive time-dependent phosphorylation patterns (Fig. 3a). To identify potential relationships between the p-site trajectories and the regulation of particular cellular functions, cell process enrichment was compared across groups. The analysis revealed the rapid and broad consequences of glucose stimulation on the phospho-proteome in insulin secreting cells (Fig. 3c). Early phospho-proteome remodeling included proteins involved in the regulation of cytoskeleton organization, signal transduction, transcriptional/translational regulation of gene expression and membrane trafficking along the secretory pathway, membrane recycling and exocytosis. Most of these proteins remain regulated over the first 30 and 60 min (Fig. 3b). Proteins involved in carbohydrate metabolism, energy homeostasis and Ca^2+^ transport were mostly regulated at the later time-points after initiation of glucose stimulation (30 and 60 min). Theses cellular processes may be required for the gradual long-term nutrient activation of beta-cells associated with a net increase of insulin secretion.

In addition to the systematic analysis of phosphorylation changes as described above, we also specifically focused on categories of effector proteins involved in metabolism, calcium signaling, protein (insulin) translation, regulation of the cytoskeleton, insulin trafficking, insulin granule biogenesis and exocytosis. All these biological processes are closely linked to the secretion of insulin and its regulation (Fig. 4). Included were those phosphorylation sites with a fold change (Log2) compared to control > 0.3 or < − 0.3. In this selection of p-sites, the large majority of significant phosphorylation changes already occurred at 5 min revealing rapid glucose-induced activation of signal transduction. The exception was metabolism. The few post-translationally modified metabolic enzymes were phosphorylated for most part only after 30 min in the presence of glucose. The relevance of several phosphoproteins for beta-cell functions in each of the biological processes has been already summarized in the result section.

The phosphorylation status of individual p-sites is determined by the activity of a subset of kinases and phosphatases whose relationships are annotated in a number of open access databases. This wealth of information makes it possible to deduce kinase activities from phosphoproteomic data sets. Here we used KSEA to calculate changes in kinase and phosphatatase activities based on our collective p-site results following stimulation of INS-1E cells with glucose. There are two strong arguments in favor of the usefulness of this approach to predict kinase and phosphatase activities. First, we observed a good agreement between KSEA and Western blotting results. Phosphorylation of substrates that were used to predict the activity of kinases in the MAP kinase pathway, PKC family, PKA, Akt, AMPK and Eef2K follow a time-course similar to the time-dependent phosphorylation of individual substrates revealed using p-site specific antibodies. Second, many of the results obtained by KSEA confirm earlier published work on individual signaling pathways and their regulation in beta**-**cells. By extension, KSEA may predict the activation of signal transduction pathways less well understood in beta-cells. We conclude KSEA is a powerful tool to understand short-term glucose dependent regulation of kinase and phosphatases in insulin secreting cells. It should be mentioned however that the limited p-site coverage in our study (3085 p-sites) and the dependence of our work on rat cells (INS-1E), whose kinase-substrate databases are less annotated, reduced the power of this analysis [35].

The contribution of different glucose-stimulated kinases to acute glucose-induced insulin secretion are still debated. PKC, PKA and ERK1/2 activities have been shown to promote insulin secretion mainly through the regulation of the cytoskeleton and the activation of the last steps in insulin granule exocytosis [19]. In our study, the most strongly inhibited kinase following glucose stimulation is EEF2K. Rapid p-site dephosphorylation of Eef2 (T57) observed in our proteomic dataset was confirmed by Western blotting. This regulation is essential for glucose dependent stimulation of protein translation. NUAK1 was the second most inhibited kinase activity detected with KSEA. NUAK1 is a member of the AMPK-related protein kinase family, and an important regulator of cell senescence, adhesion, migration and cellular metabolism In vitro, its activity is positively regulated by increasing levels of AMP [58]. During glucose stimulation, AMP concentrations rapidly decline explaining inhibition of NUAK1 and AMPK. Results with muscle specific KO mice suggest that NUAK1 suppresses glucose uptake through negative regulation of insulin signaling in oxidative muscle [59]. To date, NUAK1 has not been studied in pancreatic beta-cells. Whether acute and/or chronic regulation of AMPK itself has an impact on insulin secretion is not clear [24, 60–62]. KSEA also points to the glucose regulation of a number of phosphatases. Calcineurin was found to be activated, consistent with reports from the literature. Furthermore, KSEA predicted dual-specificity phosphoprotein phosphatases (DUSPs) to be strongly and rapidly inhibited following glucose stimulation. In vitro*,* DUSPs inactivate mitogen-activated protein (MAP) kinase by dephosphorylation.

A second objective of this study was to identify links between signal transduction and mitochondrial energy metabolism. Glucose primarily stimulates mitochondria through the provision of substrates causing an almost immediate increase of respiration followed by a gradual increase of respiration over a time course of 5–60 min. This second phase after glucose addition depends almost completely on calcium signaling. Here we tested whether in addition to calcium other signaling pathways associated with glucose stimulation are able to modulate the mitochondrial respiratory response to the nutrient. We hypothesized that glucose regulated-kinases may have mitochondrial protein substrates that could link cytosolic signal transduction to mitochondrial activity. However, in our phospho-proteome dataset, we found only two proteins in the Mitocarta whose phosphorylation status was significantly changed following glucose stimulation: Elac2 _S800_ and Phyhipl _S15_. Elac2 is an endonuclease removing 3′ nucleotides from tRNA precursor molecules. Phyhipl stands for phytanoyl-CoA hydroxylase-interacting protein-like. Neither protein suggests an obvious link to the short-term regulation of mitochondrial respiration by glucose.

In order to test whether any of the signal transduction pathways associated with glucose stimulation predicted with KSEA impacts on the mitochondrial respiratory response, we pharmacologically manipulated key signaling pathways. Compounds were selected to target mTOR, MEK1/2, PI3kinase, p38MAPK, AMPK, Cam-kinase, calcineurin, cAMP levels, PKA and PKC. The majority of the 27 tested compounds (each compound was tested at three different concentrations) had no acute effect on glucose-induced respiration. The exceptions were inhibitors of the three kinases PKC, Cam-kinase and PI3K, which significantly lowered acceleration of respiration by glucose. The data with the PKC inhibitors confirmed our earlier findings demonstrating that the PKC inhibitor Go-6983 is able to lower the glucose induced respiratory response, while activation of PKC in the absence of stimulatory glucose is able to augment respiration [28]. The CamK-II inhibitor KN62 also caused a consistent reduction of glucose-induced respiration. These results are consistent with previous reports [63, 64]. KN62 was found to impair Ca^2+^ signaling strongly reducing depolarization-induced cytosolic calcium rises. Inhibition of respiration is therefore likely the consequence of lowered calcium signals in the cytosol and as a consequence the mitochondrial matrix. Preventing mitochondrial Ca^2+^ rises is known to inhibit glucose-induced respiration [11]. Surprisingly, KN93, an alternative inhibitor of CamK II failed to inhibit the glucose respiratory response although it was shown previously to affect calcium signaling similar to KN62. Whether and how CamK II affects mitochondrial function requires further experiments.

Interestingly, the PI3kinase inhibitor wortmanin lowered glucose-induced respiration. PI3 kinase is an essential lipid kinase activated during insulin receptor signaling. PI3 kinase activity is restricted to the plasma membrane and acts upstream of Akt/PKB. In beta-cells, insulin signaling is initiated following glucose stimulation as insulin secretion leads to rapid autocrine activation of the insulin receptor. It is therefore not surprising that our KSEA analysis identified Akt among the rapidly activated kinases (Fig. 5a and Additional file 14: Figure S6). Among the proteins phosphorylated by Akt are well-established mitochondrial substrates such as Bad or Bcl-xl, and Akt can even accumulate into the mitochondria [65]. Such Akt substrates may mediate the stimulatory effect of insulin receptor signaling on mitochondrial respiration. A second possible explanation for the activation of mitochondrial respiration by Akt is via stimulation of glycolysis. Akt activation has been shown in a number of cell types to increase glycolytic rates through regulation of glucose transport, hexokinase and phosphofructokinase activity. Whatever the exact mechanism, our results show that insulin signaling contributes to the acute regulation of mitochondrial activity during glucose stimulation.

Our pharmacological study also provides a number of valuable information on signaling pathways not regulating the acute respiratory response. Among the many compound used to manipulate cAMP and PKA signaling none altered glucose-induced respiration. Furthermore, acute inhibition or activation of AMPK did not influence respiration in INS-1E cells. The two calcineurin inhibitors cyclosporine A and FK506 also had no effect on the respiratory response to glucose. Lastly, the mTORC1 inhibitor rapamycin did not modified the glucose-dependent respiratory response (Fig. 6a). Short-term regulation of these kinases and phosphatases does not likely influence mitochondrial function in beta-cells.

## Conclusions

Glucose stimulation quickly remodels the beta-cell phosphoproteome over the first hour in presence of the nutrient, affecting a large number of biological processes linked to insulin biosynthesis, early membrane trafficking, insulin granule biogenesis and transport, exocytosis and cytoskeleton dynamics.

The present study provides us with a wealth of information regarding the kinetics and the activation and inhibition of kinases and phosphatases and paves the way to study their contribution to the regulation of beta-cell function.

Our study also gives new insight into beta-cell signaling and interactions with glucose-induced metabolic activation of mitochondria, a key cellular event for the transition from a resting to an activated beta-cell. We observe that three nutrient activated kinases: phosphoinositide 3-kinase, Ca2+/calmodulin dependent protein kinase and protein kinase C crosstalk to mitochondrial energy metabolism.

## Additional files

Additional file 1: **Figure S1.** Randomization of the samples and conditions for the proteomic analysis. TMT labelling was performed as indicated with the code of colors. Stimulation with PMA was also included in the experiments but the results were previously reported [28]. We present here the results of the glucose stimulation for the time series. (PDF 40 kb)

Additional file 2: **Figure S2.** Distribution of phosphosites per protein. (PDF 4 kb)

Additional file 3: **Table S1.** Significantly regulated p-sites upon 5 min of glucose stimulation. The table shows p-site quantification of five experimental replicates performed on both experimental conditions (2.5 and 16.7 mM glucose), including delta differences and *p*-values. The table displays only regulated p-sites with *p*-value ≤0.05 (unpaired t-test) and FC (Log2) > 0.3 or < − 0.3, and includes information on the p-sites identified and quantified, comprising localization probability, peptide sequence and protein accession number (Uniprot KB). (XLSX 29 kb)

Additional file 4: **Table S2.** Significantly regulated p-sites upon 30 min of glucose stimulation. The table shows p-site quantification of five experimental replicates performed on both experimental conditions (2.5 and 16.7 mM glucose), including delta differences and *p*-values. The table displays only regulated p-sites with *p*-value ≤0.05 (unpaired t-test) and FC (Log2) > 0.3 or < − 0.3, and includes information on the p-sites identified and quantified, comprising localization probability, peptide sequence and protein accession number (Uniprot KB). (XLSX 37 kb)

Additional file 5: **Table S3.** Significantly regulated p-sites upon 60 min of glucose stimulation. The table shows p-site quantification of five experimental replicates performed on both experimental conditions (2.5 and 16.7 mM glucose), including delta differences and p-values. The table displays only regulated p-sites with *p*-value ≤0.05 (unpaired t-test) and FC (Log2) > 0.3 or < − 0.3, and includes information on the p-sites identified and quantified, comprising localization probability, peptide sequence and protein accession number (Uniprot KB). (XLSX 48 kb)

Additional file 6: **Figure S3.** Glucose-dependent regulated proteins. A) Volcano plots displaying the distribution of significant regulated proteins overtime. Proteins significantly changed (*p*-value < 0.05) and undergoing FC (Log2) > 0.3 or < − 0.3 compared to control are shown in color. B) Volcano plots displaying the distribution of proteins containing significant regulated p-sites overtime. C) Distribution of p-sites versus their protein levels overtime. (PDF 351 kb)

Additional file 7: **Table S4.** Identification of proteins containing glucose regulated p-sites and concomitantly regulated at the protein level. The table shows the effect of glucose on the protein level of proteins containing glucose-regulated p-sites, and includes information on the fold change, *p*-value, accession number, and the number / percentage of regulated proteins. (XLSX 37 kb)

Additional file 8: **Table S5.** Gene ontology localization enrichment on significantly regulated phospho-proteins after 5, 30 and 60 min of glucose stimulation. Phospho-regulated proteins IDs (Uniprot KB) were matched to the corresponding genes. Subsequently genes were assigned to gene ontologies using Metacore®. The table displays a cell process enrichment analysis (p-value and false discovery rate, FDR) and the list of genes significantly regulated in every particular category. (XLS 415 kb)

Additional file 9: **Table S6.** Gene ontology cell process enrichment on significantly regulated phospho-proteins after 5, 30 and 60 min of glucose stimulation. Phospho-regulated proteins IDs (Uniprot KB) were matched to the corresponding genes. Subsequently genes were assigned to gene ontologies using Metacore®. The table displays a cell process enrichment analysis (p-value and false discovery rate, FDR) and the list of genes significantly regulated in every particular category. (XLS 2754 kb)

Additional file 10: **Table S7.** Gene ontology cell process enrichment on phospho proteins exclusively regulated either at 5, 30 or 60 min. Phospho-regulated proteins IDs (Uniprot KB) were matched to the corresponding genes. Subsequently genes were assigned to gene ontologies using Metacore®. To identify the most differentially affected categories between groups, the standard deviation of the –Log10 (*p*-value) amongst the three groups was calculated for every GO term and then the whole ontology was sorted in decrease order of standard deviation. The table displays a cell process enrichment analysis (*p*-value and false discovery rate, FDR) and the list of genes significantly regulated in every particular category. (XLS 2006 kb)

Additional file 11: **Figure S4.** Gene Ontology enrichment analysis in phosphoproteins exclusively regulated either at 5, 30 or 60 min. Heatmap displaying the top 30 differentially enriched ontology terms overtime considering proteins containing p-sites exclusively regulated at specific time points. (PDF 1764 kb)

Additional file 12: **Table S8.** List of genes assigned to different trajectory clusters and used for gene ontology analysis. (XLSX 9 kb)

Additional file 13: **Figure S5.** Kinase-substrate enrichment analysis upon 5, 30 and 60 min of continuous glucose stimulation. A) Heatmaps containing KSEA scores for every experimental replicate at 5, 30 and 60 min (from left to right). For kinases higher KSEA positive scores (in red) indicates higher activity whereas negative scores (in blue) indicates lower activity. Conversely, for phosphatases higher KSEA positive scores (in red) indicates lower activity whereas negative scores (in blue) indicates higher activity. The statistical significance of the KSEA score was evaluated, *p*-value (*** *p* < 0.001; ** p < 0.01*;* * *p* < 0.05). B) Average of KSEA scores were calculated for every time point and displayed in a heatmap after hierarchical clustering. (PDF 830 kb)

Additional file 14: **Figure S6.** KSEA output robustness. Groups of p-sites defining the regulation of specific kinases during glucose stimulation are shown in A-F. Changes p-status are plotted as changes over time. The average changes calculated from within these groups reflect the regulation of the corresponding kinases (colored lines). To confirm the signaling events revealed by KSEA, we assessed the phosphorylation status of a number of kinase substrates using phospho specific antibodies (A-F). INS-1E protein lysates were prepared at specific time-points over a time course of 0–60 min of glucose activation. The abundance of the total protein was not affected by glucose over the time window studied here as demonstrated using antibodies recognizing the unmodified proteins. For all phospho-specific antibodies tested, Western blotting analysis and quantitative phospho-proteomics agree regarding the changes in phosphorylation status following glucose stimulation. (PDF 485 kb)

Additional file 15: **Figure S7.** List of kinase activities according to its temporal trajectories. Positive and negatively regulated kinases and phosphatases were clustered in three groups according with the temporal regulation. T1 (5 min) = Early, T2 (30 min) = Intermediate and T3 (60 min) = Late. (PDF 139 kb)

Additional file 16: **Table S9.** List of drugs tested for their ability to regulate mitochondrial respiration induced by glucose. The table displays information on the drug tested, targeted mechanism, effect of the interaction and the range of concentrations tested. (XLSX 9 kb)

Additional file 17: **Figure S8.** Effect of H89 and Phenformin on basal and glucose stimulated mitochondrial respiration. Oxygen consumption recordings in INS-1E cells. (PDF 166 kb)

## Abbreviations

ER: Endoplasmic Reticulum
FC: Fold Change
FCS: Fetal Calf Serum
GO: Gene Ontology
KSEA: Kinase Substarte Enrichment Analysis
MAPK: Mitogen Activated Protein Kinase
MS: Mass Spectrometry
OCR: Oxigen Consumption Rates
PKA: Protein Kinase A
PKC: Protein Kinase C
p-site: Phosphorylation site
PTM: Post Translational ModificationKRBH: Krebs-Ringer Bicarbonate Hepes
RP-LC: Reversed-Phase Liquid Chromatography
SOCE: Store Operated Ca^2+^ Entry
