# Supplementary material for: Glucose-dependent phosphorylation signaling pathways and crosstalk to mitochondrial respiration in insulin secreting cells
Source: Cell Commun Signal. 2019 Feb 20;17:14. doi: 10.1186/s12964-019-0326-6 (PMC6381748; doi:10.1186/s12964-019-0326-6)
Supplement: Supplementary file 1 — Figure S1. Randomization of the samples and conditions for the proteomic analysis. TMT labelling was performed as indicated with the code of colors. Stimulation with PMA was also included in the experiments but the results were previously reported [28]. We present here the results of the glucose stimulation for the time series. (PDF 40 kb) [file 12964_2019_326_MOESM1_ESM.pdf]

A = Before Stimulation
B = Glucose 2.5mM_5 min
C = Glucose 16.7mM_5min
D = Glucose 2.5mM_30min
E = Glucose 16.7mM_30min
F = Glucose 2.5mM_60min
G = Glucose 16.7mM_60min
H = 1uM PMA_5min
I = 1uM PMA_30min
P = Pool (150 µg)

**Exp. 1**	**A1**	**B1**	**C1**	**D1**	**E1**	**F1**	**G1**	**H1**	**I1**	**P1**
	TMT-126	TMT-127N	TMT-127C	TMT-128N	TMT-128C	TMT-129N	TMT-129C	TMT-130N	TMT-130C	TMT-131
**Exp. 2**	**B2**	**C2**	**D2**	**E2**	**F2**	**G2**	**H2**	**I2**	**A2**	**P2**
	TMT-126	TMT-127N	TMT-127C	TMT-128N	TMT-128C	TMT-129N	TMT-129C	TMT-130N	TMT-130C	TMT-131
**Exp. 3**	**C3**	**A3**	**E3**	**F3**	**G3**	**H3**	**I3**	**B3**	**D3**	**P3**
	TMT-126	TMT-127N	TMT-127C	TMT-128N	TMT-128C	TMT-129N	TMT-129C	TMT-130N	TMT-130C	TMT-131
**Exp. 4**	**D4**	**E4**	**F4**	**A4**	**H4**	**I4**	**C4**	**G4**	**B4**	**P4**
	TMT-126	TMT-127N	TMT-127C	TMT-128N	TMT-128C	TMT-129N	TMT-129C	TMT-130N	TMT-130C	TMT-131
**Exp. 5**	**E5**	**F5**	**G5**	**H5**	**I5**	**B5**	**A5**	**D5**	**C5**	**P5**
	TMT-126	TMT-127N	TMT-127C	TMT-128N	TMT-128C	TMT-129N	TMT-129C	TMT-130N	TMT-130C	TMT-131
