# Supplementary figures and images for: Glucose-dependent phosphorylation signaling pathways and crosstalk to mitochondrial respiration in insulin secreting cells
Source: Cell Commun Signal. 2019 Feb 20;17:14. doi: 10.1186/s12964-019-0326-6 (PMC6381748; doi:10.1186/s12964-019-0326-6)

**Histogram of number of phosphosites per protein**

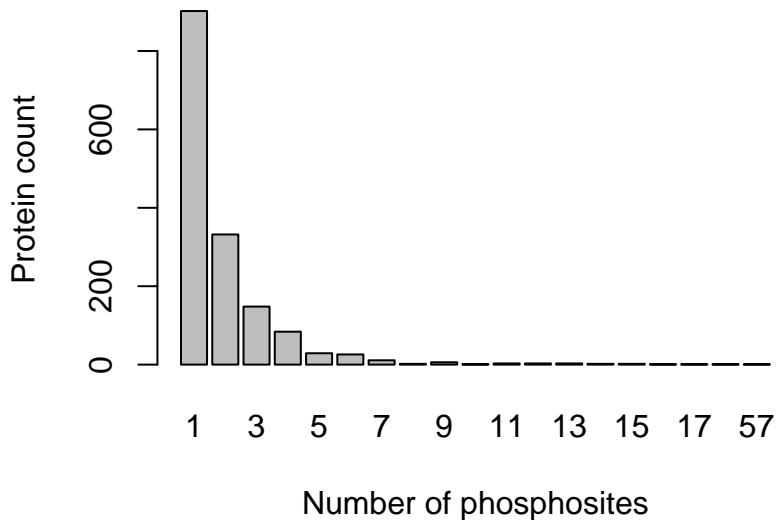

Supplement: Supplementary file 2 — Figure S2. Distribution of phosphosites per protein. (PDF 4 kb) [file 12964_2019_326_MOESM2_ESM.pdf]

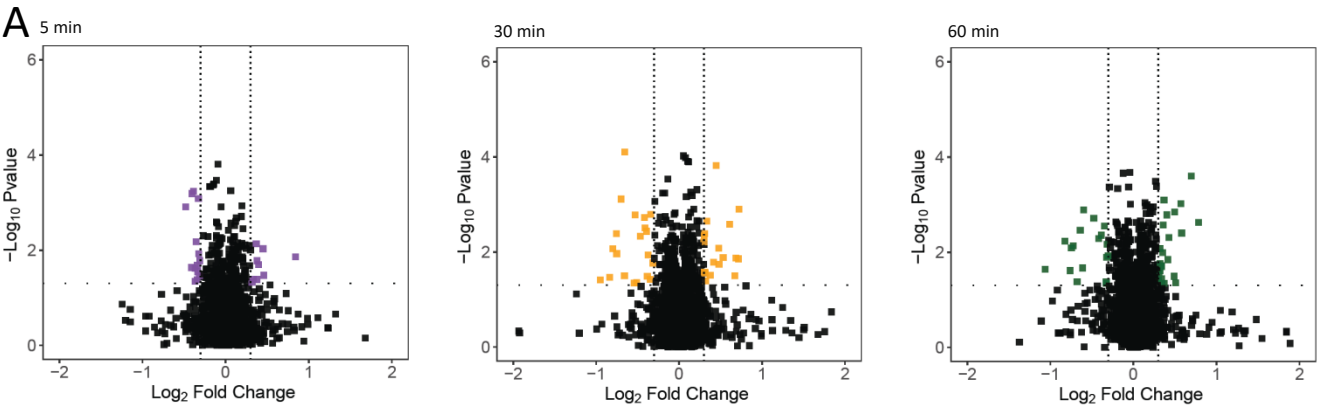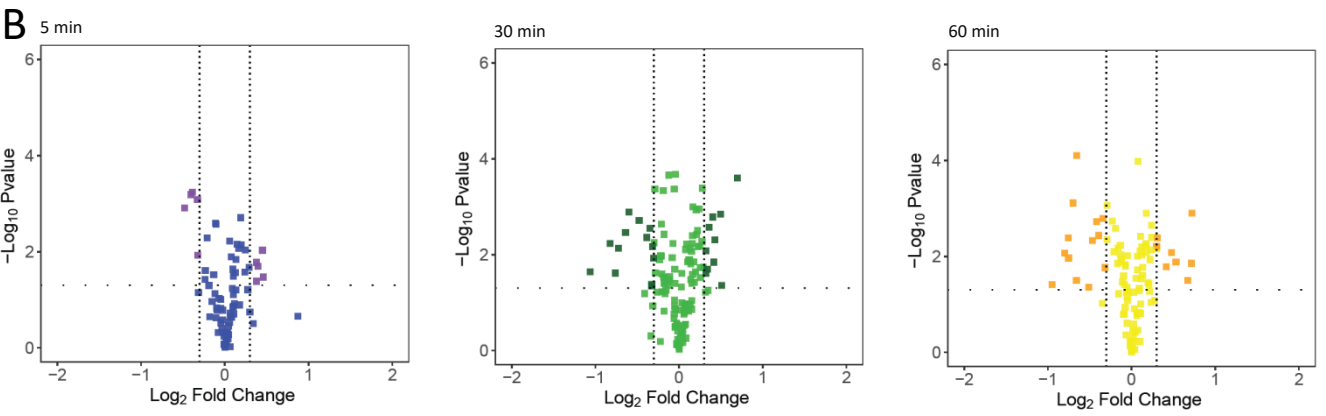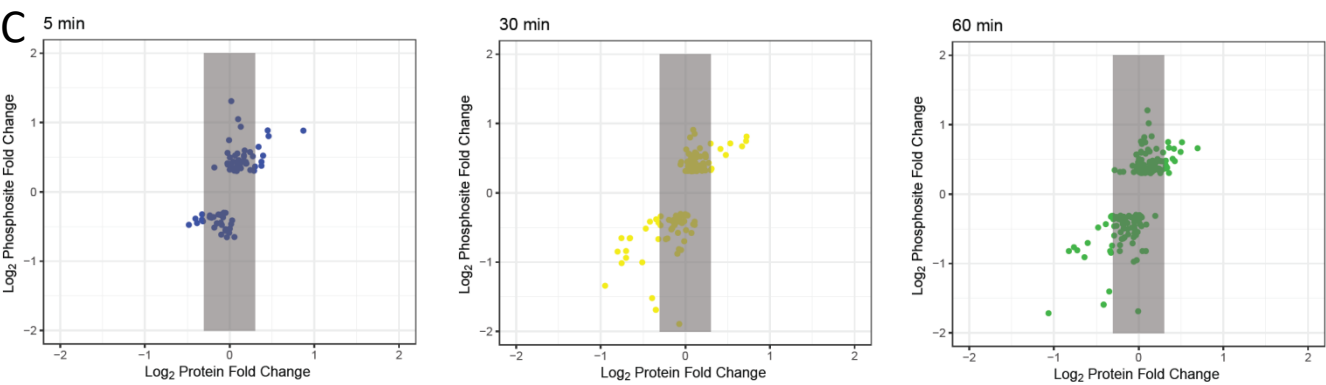

Supplement: Supplementary file 6 — Figure S3. Glucose-dependent regulated proteins. A) Volcano plots displaying the distribution of significant regulated proteins overtime. Proteins significantly changed (p-value < 0.05) and undergoing FC (Log2) > 0.3 or < − 0.3 compared to control are shown in color. B) Volcano plots displaying the distribution of proteins containing significant regulated p-sites overtime. C) Distribution of p-sites versus their protein levels overtime. (PDF 351 kb) [file 12964_2019_326_MOESM6_ESM.pdf]

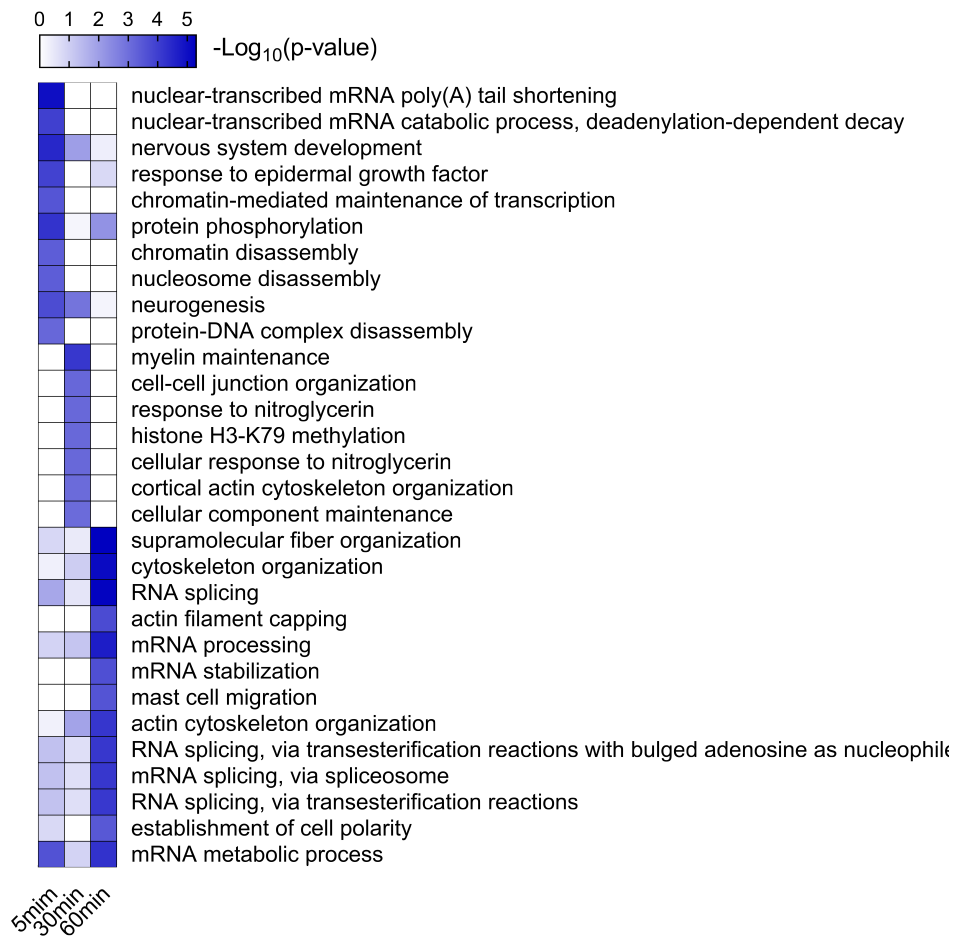

Supplement: Supplementary file 11 — Figure S4. Gene Ontology enrichment analysis in phosphoproteins exclusively regulated either at 5, 30 or 60 min. Heatmap displaying the top 30 differentially enriched ontology terms overtime considering proteins containing p-sites exclusively regulated at specific time points. (PDF 1764 kb) [file 12964_2019_326_MOESM11_ESM.pdf]

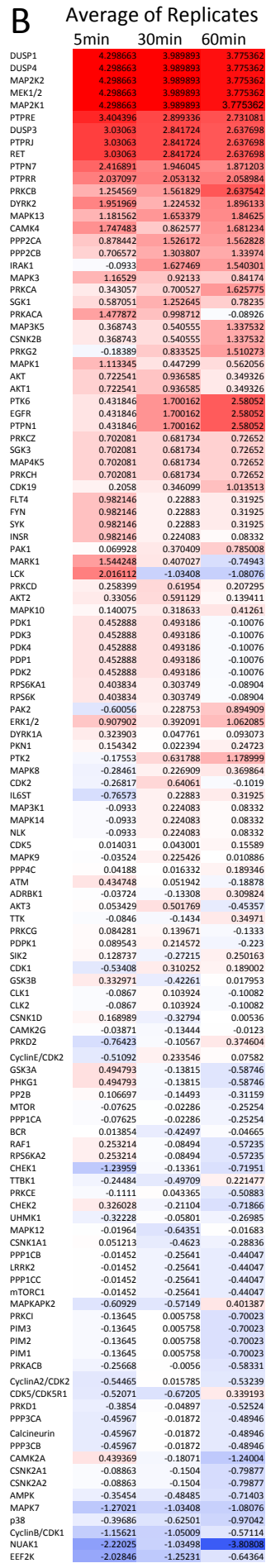

Supplement: Supplementary file 13 — Figure S5. Kinase-substrate enrichment analysis upon 5, 30 and 60 min of continuous glucose stimulation. A) Heatmaps containing KSEA scores for every experimental replicate at 5, 30 and 60 min (from left to right). For kinases higher KSEA positive scores (in red) indicates higher activity whereas negative scores (in blue) indicates lower activity. Conversely, for phosphatases higher KSEA positive scores (in red) indicates lower activity whereas negative scores (in blue) indicates higher activity. The statistical significance of the KSEA score was evaluated, p-value (*** p < 0.001; ** p < 0.01; * p < 0.05). B) Average of KSEA scores were calculated for every time point and displayed in a heatmap after hierarchical clustering. (PDF 830 kb) [file 12964_2019_326_MOESM13_ESM.pdf]

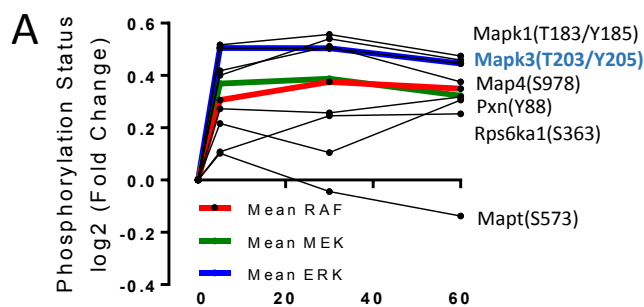

#### RAF/MEK/ERK Group

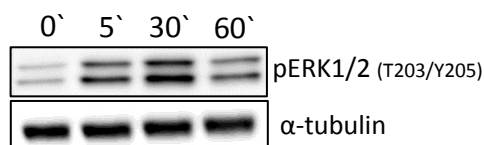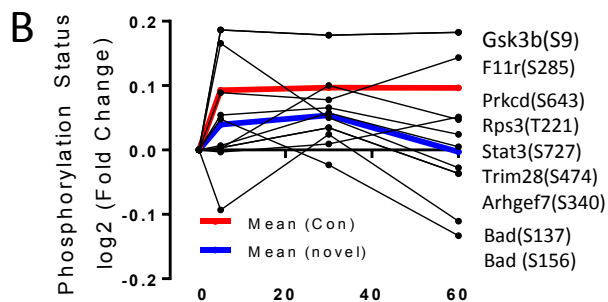

#### PKC Group

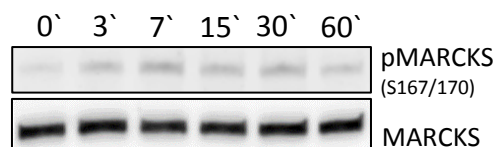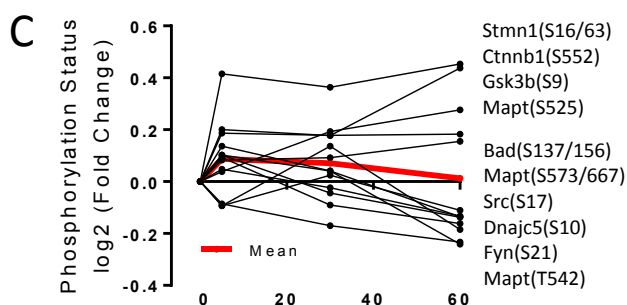

#### PKA Group

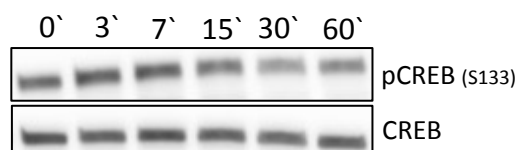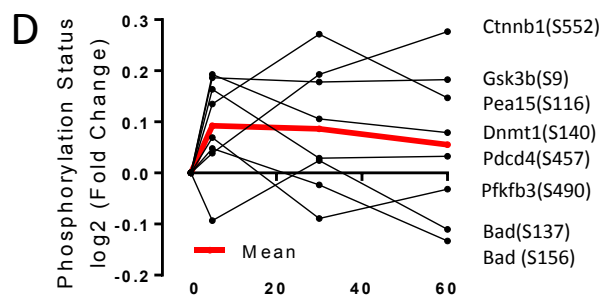

#### AKT Group

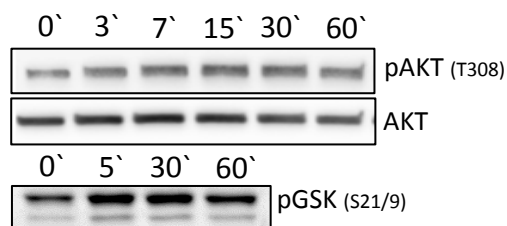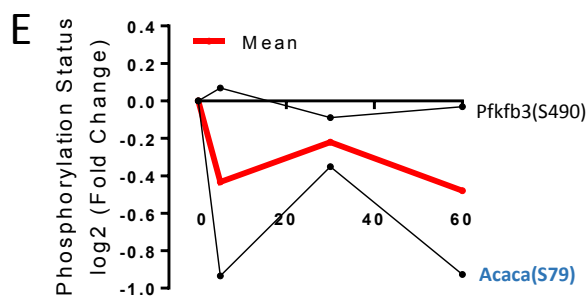

#### AMPK Group

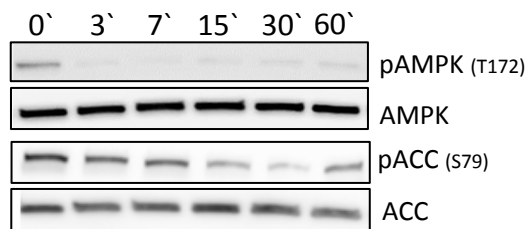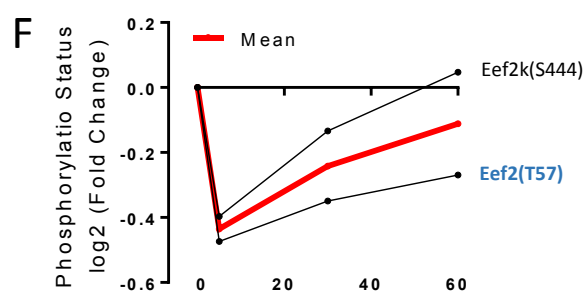

#### Eef2K Group

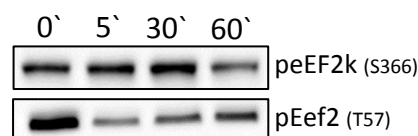

Supplement: Supplementary file 14 — Figure S6. KSEA output robustness. Groups of p-sites defining the regulation of specific kinases during glucose stimulation are shown in A-F. Changes p-status are plotted as changes over time. The average changes calculated from within these groups reflect the regulation of the corresponding kinases (colored lines). To confirm the signaling events revealed by KSEA, we assessed the phosphorylation status of a number of kinase substrates using phospho specific antibodies (A-F). INS-1E protein lysates were prepared at specific time-points over a time course of 0–60 min of glucose activation. The abundance of the total protein was not affected by glucose over the time window studied here as demonstrated using antibodies recognizing the unmodified proteins. For all phospho-specific antibodies tested, Western blotting analysis and quantitative phospho-proteomics agree regarding the changes in phosphorylation status following glucose stimulation. (PDF 485 kb) [file 12964_2019_326_MOESM14_ESM.pdf]

A

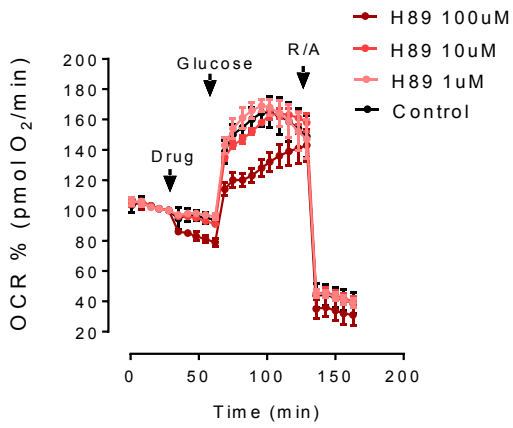

B

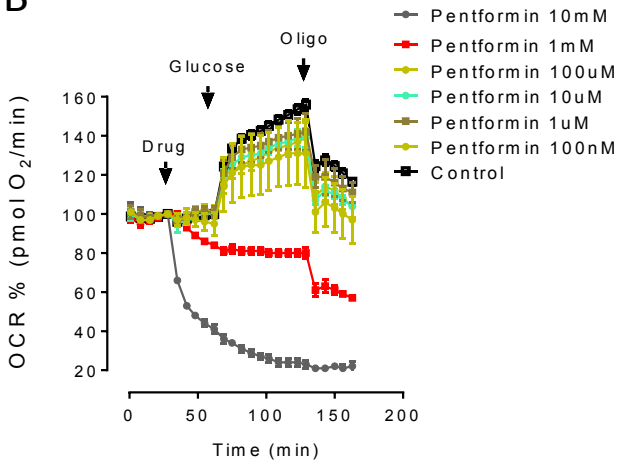

Supplement: Supplementary file 17 — Figure S8. Effect of H89 and Phenformin on basal and glucose stimulated mitochondrial respiration. Oxygen consumption recordings in INS-1E cells. (PDF 166 kb) [file 12964_2019_326_MOESM17_ESM.pdf]
