# Supplementary material for: Glucose-dependent phosphorylation signaling pathways and crosstalk to mitochondrial respiration in insulin secreting cells
Source: Cell Commun Signal. 2019 Feb 20;17:14. doi: 10.1186/s12964-019-0326-6 (PMC6381748; doi:10.1186/s12964-019-0326-6)
Supplement: Supplementary file 15 — Figure S7. List of kinase activities according to its temporal trajectories. Positive and negatively regulated kinases and phosphatases were clustered in three groups according with the temporal regulation. T1 (5 min) = Early, T2 (30 min) = Intermediate and T3 (60 min) = Late. (PDF 139 kb) [file 12964_2019_326_MOESM15_ESM.pdf]

Positive KSEA Score upon glucose stimulation

T1 (5min)	T2 (30min)	T3 (60min)
DUSP1	IRAK1	CDK5/CDK5R1
DUSP4	PTK6	MAPKAPK2
MAP2K2	EGFR	PRKCA
MEK1/2	PTPN1	MAP3K5
MAP2K1	CHEK1	CSNK2B
PTPRE	PRKG2	TTBK1
DUSP3	IL6ST	CDK19
PTPRJ	CDK2	MAPK12
RET	CDK1	SIK2
PTPN7	PAK2	TTK
PTPRR	PTK2	CyclinB/CDK1
LCK	EEF2K	ADRBK1
DYRK2	CyclinE/CDK2	GSK3B
CAMK4	SGK1	PAK1
MARK1	PRKD2	PKN1
PRKACA	CyclinA2/CDK2	
PRKCB	MAPK8	
MAPK13	AKT3	
MAPK3	PRKCD	
MAPK1	MAP3K1	
FLT4	MAPK14	
FYN	NLK	
SYK	UHMK1	
INSR	MAPK9	
ERK1/2		
PPP2CA		
AKT		
AKT1		
PPP2CB		
PRKCZ		
SGK3		
MAP4K5		
PRKCH		
PDK1		
PDK3		
PDK4		
PDP1		
PDK2		
CAMK2A		
ATM		
RPS6KA1		
RPS6K		
AKT2		
DYRK1A		

Negative KSEA Score upon glucose stimulation

T1 (5min)	T2 (30min)	T3 (60min)
MAPK7	GSK3A	NUAK1
p38	PHKG1	PRKCI
AMPK	CHEK2	PIM3
	CSNK1A1	PIM2
	CSNK1D	PIM1
	BCR	CSNK2A1
	PP2B	CSNK2A2
	PPP1CB	PRKACB
	LRRK2	PRKCE
	PPP1CC	RAF1
	mTORC1	RPS6KA2
		PRKD1
		PPP3CA
		Calcineurin
		PPP3CB
		PDPK1
		PRKCG
		MTOR
		PPP1CA
		CLK1
		CLK2
